# Estimating crop parameters using Sentinel-1 and 2 datasets and geospatial field data

**DOI:** 10.1016/j.dib.2021.107408

**Published:** 2021-09-21

**Authors:** Audrey Mercier, Julie Betbeder, Julien Denize, Jean-Luc Roger, Fabien Spicher, Jérôme Lacoux, David Roger, Jacques Baudry, Laurence Hubert-Moy

**Affiliations:** a LETG Rennes UMR 6554, Université Rennes 2, Place du recteur Henri Le Moal, Rennes Cedex 35043, France; b CIRAD, Forêts et Sociétés, Montpellier 34398, France; c Ecosystems Modelling Unity, Forests, Biodiversity and Climate Change Program, Tropical Agricultural Research and Higher Education Center (CATIE), Turrialba, Cartago, Costa Rica; d Institute of Electronics and Telecommunications of Rennes IETR, UMR CNRS 6164, University of Rennes, Rennes 35000, France; e Institut Agro, UMR 0980 BAGAP, ESA, Rennes 35042, France; f Unité Ecologie et Dynamiques des Systèmes Anthropisés, UMR 7058 CNRS, Université de Picardie Jules Verne, 33 rue St-Leu, Amiens 80039, France

**Keywords:** LAI, Biomass, Phenological stages, Water content, Remote sensing, Optical and SAR satellite images, Wheat, Rapeseed

## Abstract

Crop monitoring is essential for ensuring food security in a global context of population growth and climate change. Satellite images are commonly used to estimate crop parameters over large areas, and the freely available Synthetic Aperture Radar (SAR) Sentinel-1 (S-1) and optical Sentinel-2 (S-2) images are relevant for that purpose combining high temporal resolution and high spatial resolution. For this data article, field surveys were conducted from January to July 2017 in France to sample wheat and rapeseed crop parameters during the entire crops cycle. Phenological stages were identified in 83 wheat fields and 32 rapeseed fields in Brittany and Picardy regions. Moreover, Leaf Area Index (LAI), wet biomass, dry biomass and water content were sampled in three wheat fields and three rapeseed fields in Brittany. We assigned to each field sample 10 spectral bands and 12 vegetation indices from S-2 images and two backscattering coefficients, one backscattering ratio and four polarimetric indicators from S-1 images. This dataset can be used for crop monitoring in other regions, as well as for modelling development.

## Specifications Table

**Table:** 

Subject	Agronomy and Crop Science
Specific subject area	Applied remote sensing; crop biophysical parameters
Type of data	Table Spatial data
How data were acquired	Spectral values and vegetation indices derived from S-2 images; backscattering coefficients and polarimetric indicators derived from S-1 images; LAI derived from hemispherical photographs using CAN-EYE software [5]; wet biomass, dry biomass and water content collected on the field.
Data format	Raw Analyzed
Parameters for data collection	S-1 and S-2 pixels completely included within wheat and rapeseed fields and associated crop parameters collected during the field surveys (phenological stages, LAI, wet biomass, dry biomass and water content) from January to July 2017.
Description of data collection	Field surveys were conducted from January to July 2017 to collect wheat and rapeseed crop parameters in 115 fields. Phenological stages were identified in 83 wheat fields and 32 rapeseed fields in Brittany and Picardy regions (France). LAI, wet biomass, dry biomass and water content were sampled in three wheat fields and three rapeseed fields in Brittany. As close as possible to the dates of field surveys, spectral values and vegetation indices were derived from S-2 images and backscattering coefficients and polarimetric indicators from S-1 images.
Data source location	Region: Brittany and Picardy Country: France
Data accessibility	With the article
Related research article	A. Mercier, J. Betbeder, J. Baudry, V. Le Roux, F. Spicher, J. Lacoux, D. Roger, L. Hubert-Moy, Evaluation of Sentinel-1 & 2 time series for predicting wheat and rapeseed phenological stages. ISPRS Journal of Photogrammetry and Remote Sensing. 163 (2020) 231-256. https://doi.org/10.1016/j.isprsjprs.2020.03.009

## Value of the Data

• The datasets provide crop biophysical parameters (LAI, biomass and phenological stages) during the crop cycles of wheat and rapeseed and associated S-1 and S-2 features for crop monitoring.
• The datasets can be used in many fields of research (Agronomy, Climatology, Ecology...) to analyze the relationships between crop growth and agricultural practices, climatic variables, landscape structure or species distribution.
• These datasets can be used for crop monitoring in other regions, as well as for modelling development.

## 1 Data Description

The datasets include a vector GIS shapefile (RGF93/Lambert-93 system, ESPG 2154) containing 55 polygons located in Picardy [2,3] and 60 in Brittany regions [3,4] (France). The 115 polygons correspond to 32 rapeseed fields and 83 wheat fields sampled in 2017 during one crop cycle. Five attribute tables were assigned to each sampled field, which are crop types (wheat/rapeseed), LAI and biomass (*i.e*., LAI, wet biomass, dry biomass, water content), phenological stages, S-1 features (i.e., backscattering coefficients and polarimetric indicators) and S-2 features (*i.e*., spectral values and vegetation indices). The three last table columns that are labeled “DOY”, “Region” and “ID” correspond to the acquisition dates of satellite images or crop parameters in Day Of Year (DOY) 2017, region of France (Brittany or Picardy) and field identifier, respectively. Table 1 provides a full description of the acronyms and abbreviations used in this article.

**Table 1:** List of acronyms and abbreviations.

Acronyms and abbreviations	Description	Unit
ID	Field identifier	Dimensionless
DOY	Day of year 2017	Day number
LAI	Leaf Area Index	Dimensionless
entropy_shannon_norm	Normalized shannon entropy	Dimensionless
entropy_shannon_I_norm	Normalized intensity of shannon entropy	Dimensionless
entropy_shannon_P_norm	Normalized polarization of shannon entropy	Dimensionless
span	Total scattered power	Decibel (dB)
VV	Sigma VV	Decibel (dB)
VH	Sigma VH	Decibel (dB)
VHVV	Sigma VH:Sigma VV	Decibel (dB)
Band 2	Blue band	Per ten thousand
Band 3	Green band	Per ten thousand
Band 4	Red band	Per ten thousand
Band 5	Red-edge band	Per ten thousand
Band 6	Red-edge band	Per ten thousand
Band 7	Red-edge band	Per ten thousand
Band 8	Near-infrared band	Per ten thousand
Band 8A	Near-infrared band	Per ten thousand
Band 11	Shortwave-infrared band	Per ten thousand
Band 12	Shortwave-infrared band	Per ten thousand
GNDVI	Green Normalized Vegetation Index	Dimensionless
IRECI	Inverted Red-Edge Chlorophyll Index	Dimensionless
MCARI	Modified Chlorophyll Absorption in Reflectance Index	Dimensionless
MSAVI	Modified Soil-Adjusted Vegetation Index	Dimensionless
MTCI	MERIS Terrestrial Chlorophyll Index	Dimensionless
NDI	Normalized Difference Index	Dimensionless
NDVI	Normalized Difference Vegetation Index	Dimensionless
PSSRa	Pigment Specific Simple Ratio	Dimensionless
REIP	Red-Edge Inflection Point	Dimensionless
SAVI	Soil-Adjusted Vegetation Index	Dimensionless
S2REP	Sentinel-2 Red Edge Position	Dimensionless
WDVI	Weighted Difference Vegetation Index	Dimensionless

## 2 Experimental Design, Materials and Methods

Concerning crop parameters, phenological stages were identified over the 115 field samples based on the Biologische Bundesanstalt, Bundessortenamt and Chemical industry (BBCH) scale [5]. These data were used in Mercier et al [2,3]. LAI, wet biomass, dry biomass and water content surveys were conducted on three wheat fields and three rapeseed fields in Brittany region. For this purpose, 10 digital hemispherical photographs were taken at each sampled field on each date. These data were used in Mercier et al [4]. LAI was estimated from the hemispherical photographs using CAN-EYE software [1] and averaged per date and field sample. Biomass measurements were performed in homogeneous areas (20 × 20 m) where five samples of wheat 50 cm and five rapeseed plants were collected during the field surveys. The wet biomass was directly weighed *in situ*, and the dry biomass was measured after drying the crop (oven, 65 °C, 48 h). Water content in the plant equals wet biomass minus dry biomass.

Concerning remote sensing features, S-1 and S-2 images were downloaded from the Copernicus Open Access Hub (https://scihub.copernicus.eu/). Cloud-free S-2 images used correspond to Level-2A products providing top of canopy reflectances. Twelve vegetation indices were calculated from S-2 spectral bands (Table 1). S-1 images used were acquired in Interferometric Wide (IW) swath mode and correspond to Single Look Complex (SLC) products. The backscattering coefficients (sigma VH and sigma VV) extraction process was performed using the S-1 Toolbox (http://step.esa.int/main/toolboxes/sentinel-1-toolbox/). This process includes (1) radiometric calibration, (2) speckle filtering using a Lee Refined 7 × 7 filter [6], (3) geometric corrections using Shuttle Radar Topography Mission data [7], (4) calculation of the sigma VH: sigma VV ratio and (5) conversion from linear to decibel values. The polarimetric indicators extraction process was performed using PolSARpro version 5.1.3 software [8]. This process includes (1) the extraction of a 2 × 2 covariance matrix, (2) a speckle filtering using a Lee Refined 7 × 7 filter, (3) the extraction of four polarimetric indicators (the Shannon entropy, the intensity, the degree of polarization and the span), (4) the normalization of the Shannon entropy, the intensity and the degree of polarization. Finally, all S-1 and S-2 images were projected onto the RGF93/Lambert-93 system (EPSG 2154) and resampled with bilinear interpolation to the resolution of 10 m. The median value was computed at the field scale with a negative buffer of 15 m and 10 m in Picardy and Brittany, respectively, to select only pixels fully contained within each field.

## CRediT authorship contribution statement

**Audrey Mercier:** Conceptualization, Methodology, Software, Validation, Data curation, Writing – original draft, Visualization. **Julie Betbeder:** Conceptualization, Methodology, Software, Supervision. **Julien Denize:** Resources, Data curation. **Jean-Luc Roger:** Resources. **Fabien Spicher:** Resources, Data curation. **Jérôme Lacoux:** Resources. **David Roger:** Resources. **Jacques Baudry:** Conceptualization, Writing – review & editing, Project administration, Funding acquisition. **Laurence Hubert-Moy:** Conceptualization, Supervision, Writing – review & editing, Project administration, Funding acquisition.

## Declaration of Competing Interest

The authors declare that they have no known competing financial interests or personal relationships which have, or could be perceived to have, influenced the work reported in this article.

## Appendix Supplementary materials

Image, application 1

Image, application 2

Image, application 3

Image, application 4

Image, application 5

Image, application 6

Image, application 7

Image, application 8

Image, application 9
